# Effects of two different doses of carbohydrate ingestion on taekwondo-related performance during a simulated tournament

**DOI:** 10.1186/s12970-021-00434-4

**Published:** 2021-05-27

**Authors:** Alireza Naderi, Mohammad Hossein Samanipour, Amir Sarshin, Scott C. Forbes, Majid S. Koozehchian, Emerson Franchini, Reid Reale, Erfan Berjisian, Erick P. de Oliveira, Hossein Miraftabi, Maryam Safari Sharafshadeh, Sajjad Rezaei

**Affiliations:** 1 Department of Sport Physiology, Boroujerd Branch, Islamic Azad University, Boroujerd, Iran; 2 Department of Sport Science, Imam Khomeini International University, Qazvin, Iran; 3 Department of Exercise Physiology, Karaj Branch, Islamic Azad University, Karaj, Iran; 4 Faculty of Education, Department of Physical Education Studies, Brandon University, Brandon, MB R7A6A9 Canada; 5 Department of Kinesiology, Jacksonville State University, Jacksonville, AL 36265 USA; 6 School of Physical Education and Sport, University of São Paulo (USP), São Paulo, Brazil; 7 UFC Performance Institute, Shanghai, China; 8 Department of Exercise Physiology, Faculty of Physical Education and Sport Sciences, Tehran, Iran; 9 Laboratory of Nutrition, Exercise and Health (LaNES), School of Medicine, Federal University of Uberlandia (UFU), Uberlandia, Minas Gerais Brazil; 10 Ebne Sina Sports Medicine Group, Elmi-Karbordi University, Tehran, Iran; 11 College of Health and Human Sciences, Charles Darwin University, Darwin, Australia

**Keywords:** High-intensity intermittent Exercice, Rating of perceived exertion, Blood glucose, Combat sports

## Abstract

**Background:**

Carbohydrate (CHO) ingestion enhances exercise performance; however, the efficacy of CHO intake on repeated bouts of exercise simulating a taekwondo tournament is unknown. Therefore, the purpose was to compare the effects of two different doses of CHO on a sports-specific kicking test during a simulated taekwondo tournament compared to placebo (PLA).

**Methods:**

In a double-blind, randomized-placebo controlled, cross-over trial, eleven junior male professional taekwondo athletes (age: 16 ± 0.8 years, body mass: 55.3 ± 7.3 kg) ingested one of three solutions: (i) high dose (C45): 45 g of CHO (60 g∙h^− 1^), (ii) low dose (C22.5): 22.5 g of CHO (30 g∙h^− 1^; both solutions containing 2:1 glucose:fructose), or a PLA immediately following each kicking test. The kicking test was repeated 5 times, separated by 45 mins of rest, simulating a typical taekwondo competition day. Ratings of perceived exertion (RPE) and gastrointestinal discomfort (GI) scores were collected immediately after, and blood glucose before each test.

**Results:**

The results revealed that C45 and C22.5 did not improve total, successful, or percentage of successful kicks compared to PLA (*p* > 0.05). Blood glucose was significantly higher following both CHO conditions compared with PLA across all five tests (*p* < 0.05). There were no differences between treatments or across tests for RPE (*p* > 0.05).

**Conclusion:**

CHO intake, independent of the dose, did not alter taekwondo kick performance during a simulated taekwondo tournament.

## Introduction

Taekwondo is one of the most popular weight-classified striking combat sports that has progressed into modern-day Olympics Games [1]. Taekwondo competition matches include three 2-min rounds, with 1 min rest between each round [2]. This combat sport is characterized by high-intensity intermittent activity with 0.7 to 1.7-s attacks that include 1–3 kicks and punches, which are interspersed with 2–8 s of low-intensity action composed by step or pause [2]. Each athlete in a tournament typically fights 3–7 matches across 1–2 days, depending on their successful advancement throughout the tournament [3]. One of the critical physiological challenges of a taekwondo (and other combat sport) athlete is the short recovery time between each match, which is typically between 30 min and 2 h during a tournament [4, 5]. As such, nutritional strategies that accelerate acute recovery between matches may potentially improve subsequent performance [4, 6, 7].

Although oxidative metabolism is the primary energy source during a taekwondo match, increased non-oxidative (i.e., anaerobic) contribution is involved during the kicking and punching actions, and is required to maintain lower-limb power output [2]. Carbohydrate (CHO) in the form of muscle and liver glycogen is the main finite fuel to regenerate ATP via both anaerobic and oxidative metabolic pathways [8]. Lower blood glucose and CHO oxidation rates along with glycogen storage depletion during prolonged exercise contribute to fatigue [9], which may impair exercise performance [10, 11]. CHO ingestion before and/or during exercise in a dose/type-dependent manner can prevent hypoglycemia and sustain exercise performance in both continuous and intermittent high-intensity prolonged exercises [12–15]. However, CHO’s ergogenic benefits depend on the duration, intensity, and type of exercise [16]. CHO (30–90 g∙h^− 1^) containing a 2:1 ratio of glucose:fructose are recommended for exercise lasting 1–3 h [17], whereas rinsing ones mouth with a CHO solution for 10 s without ingestion may improve exercise performance at times when substrate availability is not typically performance limiting (e.g., in efforts lasting < 30 min) via central nervous system effects [17]. Further, CHO ingestion during recovery from intense exercise is critical to replenish glycogen storage and may aid subsequent exercise performance in events with short recovery times (< 4 h) [18]. Recently, McCarthy et al. 2020 showed that 1.2 g CHO∙kg^− 1^ ingested during 2 hours of recovery between repeated bouts of interval endurance exercise improved subsequent exercise performance, reduced ratings of perceived exertion (RPE) and fatigue by 35% compared to placebo [7]. The aforementioned findings highlight the impact of CHO on acute performance and suggest improvements in subsequent exercise performance in a variety of contexts when short recovery periods between bouts exist; however, the role of CHO ingestion on multiple daily matches such as a taekwondo tournament is yet to be investigated. In taekwondo athletes, greater cortisol [19] along with higher blood lactate, increases reaction time and reduces kick impact in a fatigued state [20], which may be related to glycogen depletion [21]. However, counterintuitively, no ergogenic effect of CHO mouth rinsing was found when athletes were tested six times during four weeks of Taekwondo Anaerobic Intermittent Kick Tests (TAIKT) in 27 taekwondo players in both fasted or fed states [22]. Presently, there is a paucity of research that has examined the role of CHO ingestion on taekwondo performance during a simulated tournament, with athletes competing in multiple matches across a day.

Thus, this study aimed to compare the effects of two different dosages of CHO drinks, equating to 30 and 60 g∙h^− 1^ compared to placebo, on: total kicks, successful kicks and successful kicks percentage, blood glucose (BG), and RPE following five sport-specific kicking tests repeated every 45 min, designed to simulate a taekwondo tournament. We hypothesized that CHO ingestion would augment total and successful kicks by maintaining BG across multiple tests, in a dose response fashion, compared to placebo (PLA).

## Methods

### Participant

Twelve junior male professional taekwondo black belt athletes (age: 16 ± 0.8 years, body mass: 55.3 ± 7.3 kg, height: 177.6 ± 1.9 cm, competition experience: 5.8 ± 1.0 years) were recruited to participate in this study. The inclusion criteria of the study included having more than 5 years’ experience in taekwondo competitions, and training more than 5 hours during the week for 3 months prior to the initiation of the study. Participants were excluded from the study if they had skeletal muscle injuries or reported consuming any nutritional ergogenic aids within 3 months of the start of the study. Participants were informed about the nature and potential risks and benefits of the experimental protocols. Participants were not cutting weight during the study. One participant was injured during the test and was removed from the study. The study was conducted following the Declaration of Helsinki and approved by Islamic Azad University of Boroujerd, Iran.

### Design

In a randomized, double-blind, cross-over placebo-controlled manner, participants engaged in five sessions, including a screening visit, familiarization, and three experimental trials where participants ingested drinks with different contents including two doses of CHO and placebo, each experimental condition was separated by at least 5 days. The supplementation protocol followed a double-blind manner by an independent researcher at each experimental trial. At the familiarization session, each participant completed the test protocol. Participants repeated the tests to become familiar with the audio signals. An experienced taekwondo coach provided technique corrections during the simulated taekwondo effort test (STET) to ensure proper form. All of the trials were conducted on the same day of the week and at the same time controlling for alterations in circadian rhythm. The temperature and relative humidity were controlled across the tests (~ 25 °C, ~ 70% relative humidity). Participants were asked to record their diet and activity pattern 2 days before the familiarization session and to replicate it during the three experimental trials. They were also instructed to refrain from consuming alcohol and caffeine, and to avoid strenuous training 24 h prior to each test. BG was obtained before each test using a glucometer (Maximed ExiChek II; TD-4224A, Taiwan) by lancing a warmed fingertip (Same care; ExiChek, TD-4224, Taiwan).

**Fig. 1:**
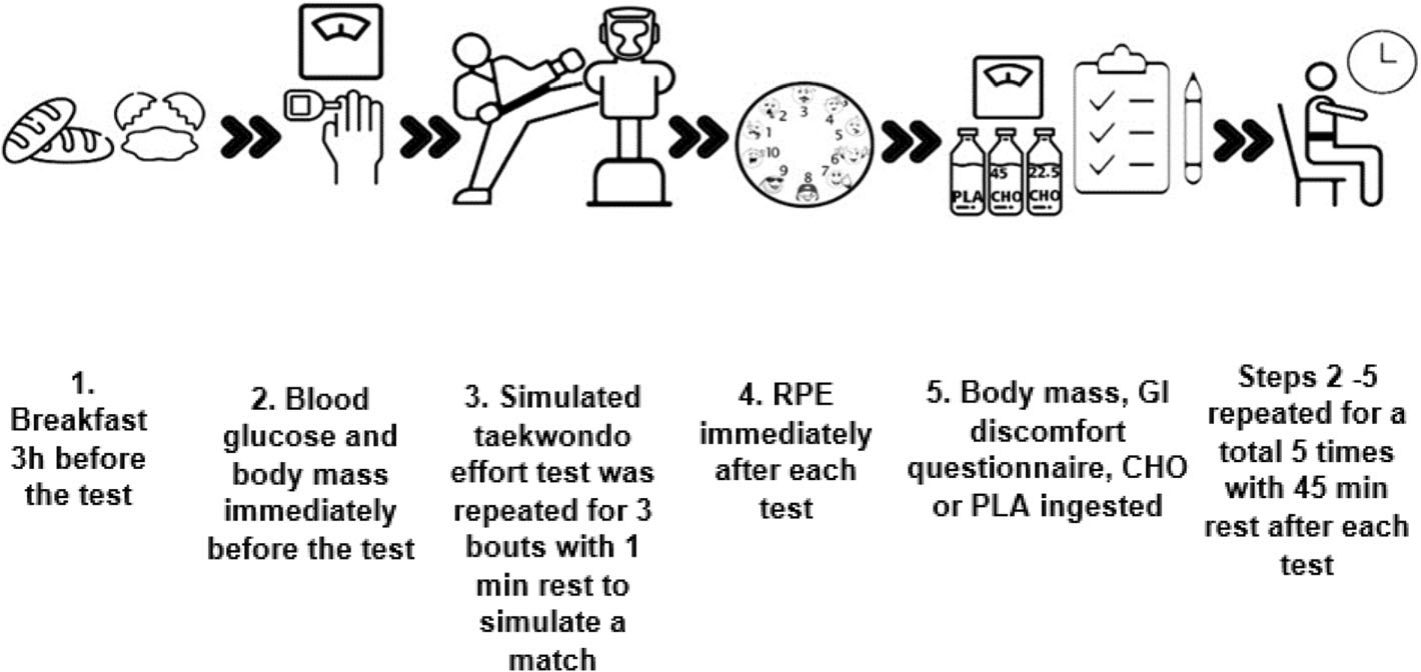
Schematic representation of the study procedures with 5 test repetitions. CHO = carbohydrate; GI = gastrointestinal; PLA = placebo

Body mass was monitored before and after each test using a calibrated scale (AFW-120 K; Seca LTD, Birmingham, United Kingdom) to investigate a possible hypohydration, which is known to decrease exercise performance [23]. A validated gastrointestinal questionnaire was also used to assess gastrointestinal (GI) distress immediately after each test [24]. A schematic of the study design is presented in Fig. 1.

### Experimental trials

Participants arrived at the gym from 7:00–7:30 a.m. each trial day and consumed a standardized breakfast consisting of ~ 1 g∙kg^− 1^ CHO (white bread) and ~ 20 g protein (6 boiled egg white). The test began 3 hours after consuming breakfast. Fifteen minutes before the first test, all participants performed a standardized warm-up that included basic kicks with a moderate rhythm and dynamic stretching. Participants were divided into four groups of three taekwondo athletes. They started to perform the STET, modified based on the Frequency Speed of Kick Test [25]. This test was divided into 3 rounds with 1 min rest between each round (each round lasting 200 s). An audio signal was used to indicate when the participants were required to start and stop kicking the ‘Hugo’ electronic dummy (HTS; Hooshmand Techno system Co, Iran) which was connected to a computer to collect data electronically automatically. Each test was repeated 5 times, separated by a 45 min rest [4] to simulate a taekwondo tournament competition day. During the STET, the stationary roundhouse (bandal-tchagui) kick, the most used taekwondo technique [25] was used. After each round, both total and successful kick number were recorded. Successful kicks were detected and recorded by an electronic foot sensor and transferred to a computer. STET data were collected using an electronic body protector (Hts & Hooshmand Techno Stem, Iran) located on the training dummy. Minimum impact thresholds were determined based on BM, age, and sex. If the kicking force was identified lower than the minimum impact threshold, the kick was not counted as a successful kick. After each test, RPE was recorded using the 6–20 Borg scale [26]. Verbal encouragement was given to all participants during the test.

### Carbohydrate beverage

In order to ensure blinding of all researchers involved in data collection during the trials, CHO beverages were prepared by an independent researcher based on 30 g∙h^− 1^ or 60 g∙h^− 1^ with glucose and fructose at a ratio of 2:1 [9, 17]. As 45 min rest intervals were applied between each test, 600 mL solutions were prepared containing either 45 g of CHO (60 g∙h^− 1^) including 30 g glucose (Roquette Industries Co., France) plus 15 g fructose (Hamburg fructose Industries Co., Germany) with 7.5% concentration or 22.5 g (30 g∙h^− 1^) CHO including 15 g glucose + 7.5 g fructose and 1.1 g sweetener powder (Sucralose E955; Kamvar, Isfahan Chocolate co., Iran) with 3.5% concentration and placebo beverage contained 2.2 g sweetener powder. Three drops of coconut flavor were added to each beverage to match the taste of all beverages. Each participant was instructed to ingest their respective beverage within 5 min after each test (tests 1 to 4), since the CHO ingestion timing pattern can influence exercise performance [27].

### Statistical analysis

Values are presented as means ± standard deviations. Normality was confirmed by using the Shapiro-Wilk test. A two-way analysis of variance with repeated measures were applied to explore the effect of different treatments (high and low CHO and PLA) and tests (5 tests over the competition day) on total kicks, total successful kicks, successful kick percentage (successful kicks/total kicks * 100), BG, and RPE. A three-way repeated measures ANOVA was applied to analyze body mass before and after each test and in each treatment. Bonferroni post hoc and within-subject 95% confidence interval (CI) error bars [28] were determined if appropriate. Partial eta-squared was used to indicate the magnitude of the differences. The interpretation of the effect size is based on the recommendations by Cohen: 0.01 = small effect size, 0.06 = moderate effect size, and 0.14 = large effect size [29]. All calculations were performed using SPSS® version 24 (IBM North America, New York, NY, USA) and the probability level for statistical significance was pre-set at *P* ≤ 0.05.

## Results

### Total kicks

**Fig. 2:**
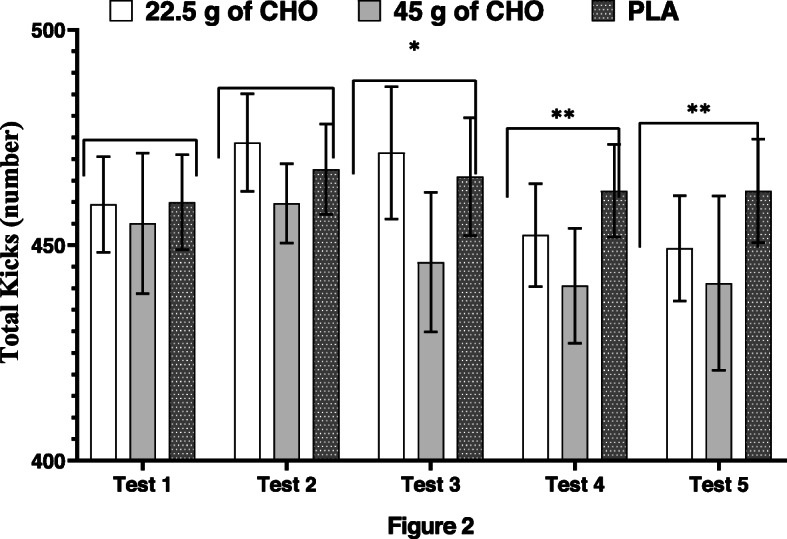
Total kicks in three treatments and five tests (values are presented as means and 95% confidence intervals). There was a significant main effect across tests for total kicks. * = significantly lower total kicks compared to tests 1 and 2 (*P* < 0.05); ** = significantly lower than tests 1, 2, and 3 (*P* < 0.05)

Figure 2 presents total kicks in each test and bouts during three treatments. For total kicks, treatment-by-test interaction (F_8,80_ = 1.126, *P* = 0.355, ${\eta}_P^2$ = 0.101) and main effect of treatment (F_2,20_ = 2.332, *P* = 0.123, ${\eta}_P^2$ = 0.189) were not significant. However, the main effect of test was significant (F4,40 = 3.698, *P* = 0.012, ${\eta}_P^2$ = 0.269), that is total kicks decreased from test one to five, as shown in Fig. 2.

### Total successful kicks

**Fig. 3:**
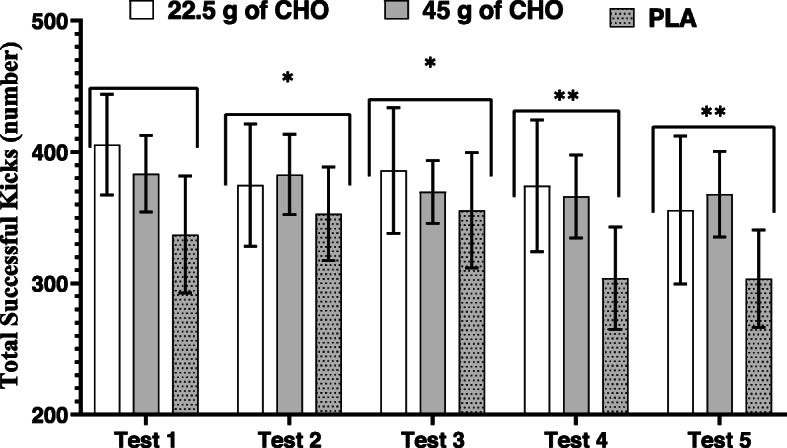
Total successful kicks in three treatments and five tests (values are presented as means and 95% confidence intervals). There was a significant main effect across tests for successful kicks. * = significantly lower total kicks compared to test 1 (*P* < 0.05); ** = significantly lower than tests 1, 2, and 3 (*P* < 0.05)

Figure 3 shows the total successful kicks in each test and bouts during the three treatments. For total successful kicks, there were no significant treatment-by-test interaction (F_8,80_ = 1.149, *P* = 0.340, ${\eta}_P^2$ = 0.103) or main effect of treatment (F_2,20_ = 2.033, *P* = 0.157, ${\eta}_P^2$ = 0.169). However, the main effect of test was significant (F_4,40_ = 3.836, *P* = 0.010, ${\eta}_P^2$ = 0.277), with total successful kicks decreasing from test one to five.

### Successful kick percentage

**Fig. 4:**
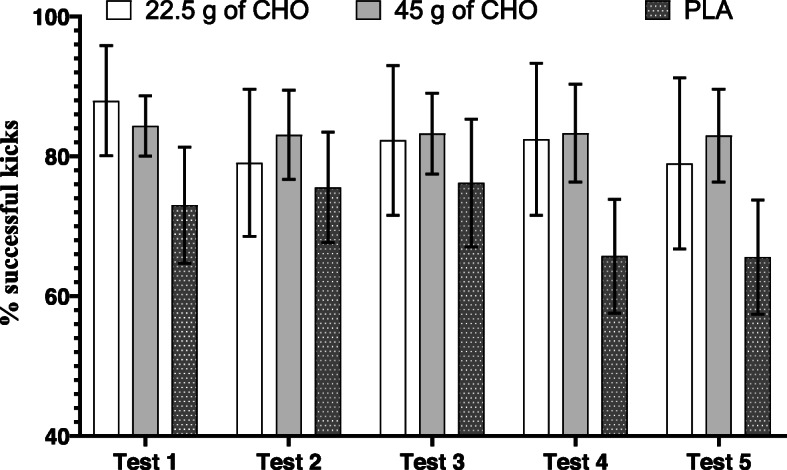
Successful kick percentage in three treatments and five tests (values are presented as means and 95% confidence intervals)

Treatment-by-test interaction (F_8,80_ = 1.401, *P* = 0.209, ${\eta}_P^2$ = 0.123), main effect of treatment (F_2,20_ = 2.927, *P* = 0.077, ${\eta}_P^2$ = 0.226), and main effect of test were not significant (F_4,40_ = 2.575, *P* = 0.052, ${\eta}_P^2$ = 0.205) for successful kick percentage (Fig. 4).

### Blood glucose

**Fig. 5:**
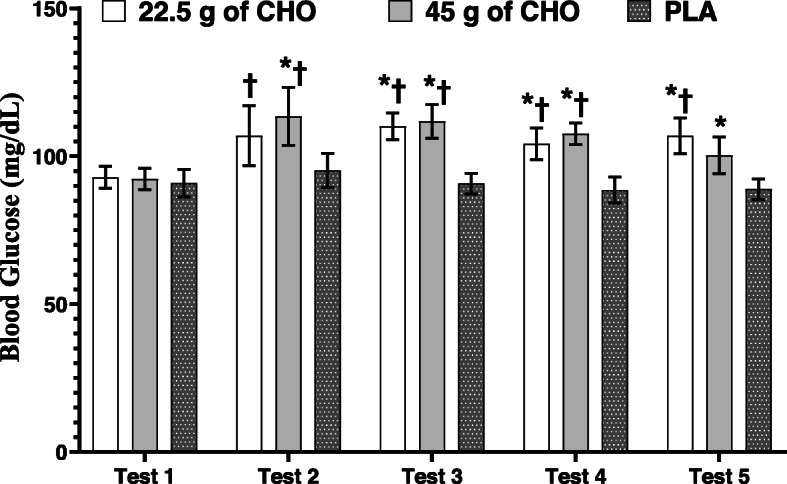
Blood glucose in three treatments and five tests (values are presented as means and 95% confidence intervals). * = significantly different than PLA (*P* ≤ 0.05). † = significantly different from test 1 in same treatment (*P* ≤ 0.05)

There was a significant treatment-by-test interaction (F_8,80_ = 2.836, *P* = 0.008, ${\eta}_P^2$ = 0.221), for BG. BG increased from test one to four in both CHO conditions (22.5 and 45 g of CHO) and decreased in test five. Results of further analysis using simple analysis (level of significance was 0.0125), within-subject standard deviation, and 95% CI are illustrated in Fig. 5.

### Rating of perceived exertion

The treatment-by-test interaction (F_8,80_ = 0.609, *P* = 0.768, ${\eta}_P^2$ = 0.057) and main effect of treatment (F_2,20_ = 0.643, *P* = 0.536, ${\eta}_P^2$ = 0.060) for RPE were not significant. The main effect of test was significant (F_4,40_ = 4.584, *P* = 0.004, ${\eta}_P^2$ = 0.314), and RPE increased from test one to five in all treatments.

### Body mass

The treatment-by-test-by-time interaction (F_8,80_ = 0.459, *P* = 0.857, ${\eta}_P^2$ = 0.047), treatment-by-time interaction (*P* = 0.108), treatment-by-test interaction (*P* = 0.092) and main effect of treatment (*P* = 0.787) for body mass was not significant. However, time-by-test interaction (*P* = 0.001), the main effect of test (*P* < 0.001) and the main effect of time (*P* < 0.001) were significant. Body mass increased from test one to five and decreased from pre-test to post-test in all treatments, but it was not different between treatments.

### Gastrointestinal symptoms

Only one participant reported GI discomfort following 45 g of CHO, reporting very severe reflux/heartburn after tests two and three and a minor one after tests four and five. As well he reported severe belching, bloating, stomach pain/cramps, vomiting, and nausea after test two, and severe stomach pain/cramps after test three.

## Discussion

To our knowledge, this is the first study to investigate the two doses of post-exercise recovery CHO intake (30 and 60 g∙h^− 1^) compared to placebo on repeated sport-specific kicking performance across a simulated taekwondo competition day. In contrast to our hypothesis, CHO intake, independent of dose, failed to enhance total kicks, total successful kicks, and percentage of successful kicks compared to placebo.

Taekwondo is characterized by repeated bursts of high-intensity activities interspersed with brief rest periods. Oxidative phosphorylation contributes the greatest amount of ATP re-synthesis (~ 58–66%); primarily to support low-intensity actions and rest intervals [2], while non-oxidative energy systems (i.e., PCr) and anaerobic glycolysis contribute ~ 26–30% and 4–5%, respectively [2, 30]. Non-oxidative phosphorylation predominantly supports explosive actions, such as kicking, punching, explosive advances, and evasions during a match [30]. Santos et al. reported that a single high-intensity action lasting 1 second was followed by 2–8 s of step or pause [30]. Further, similar to our taekwondo test, a 1:2 effort-pause ratio during a taekwondo youth Olympic match was observed [31]. As such, we modified the Frequency of Speed Kick test with 1:1 effort-pause ratio in taekwondo [25] to more closely simulate the effort-pause of a typical taekwondo match. Additionally, the use of this effort-pause ratio was able to improve Taekwondo-related performance [32].

CHO storage (i.e. muscle and liver glycogen) and oxidation play an important role during prolonged low-intensity and short duration high-intensity exercise performance [16]. CHO ingestion before and/or during exercise delays peripheral fatigue (i.e., glycogen depletion) and enhances exercise performance [16]. Ingestion of 30–90 g CHO∙h^− 1^ in a dose dependent manner enhances exercise capacity, potentially through maintenance of BG/glycogen and allowing greater CHO availability and utilization for anaerobic and aerobic glycolysis during exercise > 1 h [17]. The rate of glycogen depletion during high-intensity exercise also appears to be an important factor associated with metabolic fatigue [33]. As such, CHO ingestion during acute high intensity exercise has been shown to improve performance. For example, CHO ingestion enhanced short duration repeated sprints performance [14, 34]. However, these findings were in contrast to our results, possibly suggesting nutrient timing, duration and intensity of exercise, and recovery time intervals may modulate the ergogenic effects of CHO supplementation.

Taekwondo matches are comprised of intermittent high intensity burst of activity followed by periods of low intensity exercise or rest. During a tournament day these matches are repeated three to five times interspersed with passive rest (ranging from ~ 30 min to 3 h) [3]. We hypothesized that CHO ingestion immediately following each sport-specific kicking test would maintain kick performance and euglycemia, across repeated tests. However, in contrast to our hypothesis, there was no statistical differences for percentage of successful kicks, total successful kicks and total kicks across the five tests with either a high (60 g∙h^− 1^) or low dose (30 g∙h^− 1^) of CHO compared to PLA. Within combat sports and competition setting, there is limited evidence pertaining to CHO supplementation and performance and presently it is difficult to compare across studies due to considerable methodological differences, such as different assessments, participant’s characteristics, and various types of CHO and dosages used. Nevertheless, previous research suggests that CHO ingested during short recovery periods can augment subsequent exercise capacity and glycogen storage in endurance athletes [7, 35]. In this regards, two recent studies reported that subsequent endurance exercise was improved after 1.2 g∙kg^− 1^∙h^− 1^ CHO was ingested during a 2–4 h recovery period followed by an intermittent high intensity aerobic test [7] and time to exhaustion at 70% VO_2_max [35]. In the present study, an absolute dose of CHO was used for all participants. This dosing strategy was selected due to the relatively short recovery time and the practically of an absolute dose. Furthermore, the kick test and the repetitions of the test were selected to simulate a typical competition day, thus enhancing the external validity. In support of our results, Podlogar et al. 2020 failed to show significant improvements in subsequent exercise performance when either glucose alone or co-ingestion of glucose and fructose were compared to placebo, despite higher CHO oxidation rates following the ingestion of multiple transportable CHOs [36]. In one of the few studies utilizing taekwondo athletes, no improvements in kick performance with a CHO mouth rinsing protocol in both fed or fasted states were found [22]. Collectively, despite a likely enhancement of CHO exogenous and decreasing endogenous CHO oxidation after two doses of CHO drink [12], it appears that the test used in this study could not possibly results in enough muscle glycogen depletion which may justify the lack of kick performance improvement during five repeated intermittent kick tests with 1:1 effort-pause ratio.

In the present study BG was assessed since hypoglycemia is related to metabolic fatigue during exercise and is associated with impaired performance. Hypoglycemia is defined as a BG lower than 63 mg∙dL^− 1^ (3.5 mmol∙L^− 1^) [37]. In the current study, both CHO doses were able to significantly elevate BG assessed after each test (high dose: first test 92.27 ± 7.5 mg/dL and last test 100.27 ± 13 mg/dL; low dose: first test 92.81 ± 5.5 mg/dL and last test 106.90 ± 11.08 mg/dL) compared to placebo (*P* ≤ 0.05). However, there was no indication of hypoglycemia in the PLA condition (first test 90.90 ± 8.1 mg/dL and last test 88.81 ± 5.6 mg/dL). This avoidance of hypoglycemia was likely attributed to the standardized breakfast (1 g∙kg^− 1^ CHO) provided 3 h prior to the first test, besides the liver glycogenolysis and gluconeogenesis, which may have provided sufficient fuel to maintain the glycaemia during the exercise protocol [38]. Non-glucose substrates required for gluconeogenesis, such as glycerol and lactate are elevated during a simulated taekwondo tournament [39] due to the higher secretion of catecholamines [39] and cortisol [19]. In agreement with our finding, no cases of hypoglycemia were reported (6.1 6 ± 0.8–5.6 ± 1.7 mmol/L from first to fourth combat) in ten elite taekwondo players during a tournament across 4 matches after consuming a pre-exercise CHO meal containing 2.7 to 3.7 g∙kg^− 1^ [39].

CHO ingestion did not alter RPE compared to PLA, which is consistent with previous research [36, 40]. Lastly, a mild to severe GI discomfort adverse event was reported following the high-dose of CHO ingestion (60 g∙h^− 1^) in one participant. These events are possibly related to the lower rate of gastric emptying following high intensity exercise thorough the splanchnic blood flow decrement (41).

This study also has several limitations. First, the task used in the present study, while taekwondo-specific, did not replicate all the demands and requirements of a real match with an opponent. Further, we only assessed kick performance, which is only one technique among several used in taekwondo. In addition, more research is needed to apply less or more than 45 min of recovery time between matches, as a match in a real tournament are separated by 30 min to 3 h. Future research may consider using an accelerometer attached to wireless sensors for measuring speed and force of each kick. Further, we did not assess muscle glycogen or measure CHO oxidation, which would be of interest in future research. Also, we only used male taekwondo athletes with a relatively low sample size without performing a power analysis. Therefore, future research examining female athletes along with a priori power analysis is required. Lastly, it is worth investigating the effect of CHO supplementation after acute weight loss during a competition day on combat athletes’ performance, which is a situation that muscle glycogen is likely depleted.

In conclusion, CHO ingestion, independent of dose, failed to enhance kicking performance in a simulated competition day in trained taekwondo athletes.

## Data Availability

Data and publication materials are available from the corresponding author on reasonable request.

## Abbreviations

CHO: Carbohydrate
PLA: Placebo
STET: Simulated taekwondo effort test
RPE: Rating perceived exertion
BG: Blood glucose
TAIKT: Anaerobic Intermittent Kick Tests
CI: Confidence interval
